# Towards eco-friendly pharmaceuticals: Regulatory and policy approaches for sustainable medicines use

**DOI:** 10.1016/j.rcsop.2025.100576

**Published:** 2025-02-06

**Authors:** Ammar Abdulrahman Jairoun, Sabaa Saleh Al-Hemyari, Moyad Shahwan, Sahab Alkhoujah, Faris El-Dahiyat, Ammar Ali Saleh Jaber, Sa'ed H. Zyoud

**Affiliations:** a Health and Safety Department, Dubai Municipality, Dubai, United Arab Emirates; b Discipline of Clinical Pharmacy, School of Pharmaceutical Sciences, Universiti Sains Malaysia (USM), Pulau Pinang 11500, Malaysia; c Pharmacy Department, Emirates Health Services, Dubai, United Arab Emirates; d Centre of Medical and Bio-allied Health Sciences Research, Ajman University, Ajman 346, United Arab Emirates; e Department of Clinical Sciences, College of Pharmacy and Health Sciences, Ajman University, Ajman 346, United Arab Emirates; f Clinical Pharmacy Program, College of Pharmacy, Al Ain University, Al Ain, United Arab Emirates; g AAU Health and Biomedical Research Center, Al Ain University, Abu Dhabi, United Arab Emirates; h Department of Clinical Pharmacy & Pharmacotherapeutics, Dubai Pharmacy College for Girls, AlMuhaisanah 1, Al mizhar Dubai, United Arab Emirates; i Department of Clinical and Community Pharmacy, College of Medicine and Health Sciences, An-Najah, National University, Nablus 44839, Palestine; j Clinical Research Centre, An-Najah National University Hospital, Nablus 44839, Palestine

**Keywords:** Sustainable medicines, Sustainable pharmaceuticals, Sustainability, Environmental impact

## Abstract

**Objectives:**

The current study aimed to investigate how regulatory frameworks and policies are used to support the use of sustainable medicines within the pharmaceutical sector.

**Methods:**

The Scopus database was searched to retrieve papers. Advanced search tool of the Scopus online database was used focused on the papers that have the search query included in their titles. Data analysis incorporated bibliometric indicators like publication counts and trends, visualized through VOSviewer software version 1.6.20.

**Key findings:**

A total of 43 publications on Sustainable Medicines Use were found between 2000 and 2024. Leading countries in publication output on sustainable medicines use were United Kingdom, United States, India, Italy, Portugal, and Switzerland, indicating their collaborative relationships and publication volumes. A total of 92 institutions have been involved in research on Sustainable Medicines Use. Key institutions such as the Faculty of Engineering and the Laboratory for Process and Environmental Engineering, Lowell Center for Sustainable Production, Greiner Environmental Inc., and the University of Florence are prominently featured, indicating their significant contributions to research in this area. Key journals such as the “Journal of Cleaner Production,” “Business Strategy and the Environment,” “Chemical Engineering Transactions,” “Benchmarking,” and “Lecture Notes in Mechanical Engineering” are prominently featured. The retrieved articles have been cited an average count of 22.26. The overlay visualization created using VOSviewer suggest a shift towards exploring new drug categories, innovative approaches, and the commercial aspects of sustainability. Future research directions are likely to delve deeper into innovative methods and sustainable chemical practices (green chemistry), reflecting an emphasis on developing greener processes and products.

**Conclusion:**

This study offers a thorough analysis of the legislative and governmental strategies promoting the use of sustainable medicine. It offers important insights for promoting sustainability in the pharmaceutical industry by pointing out gaps, defining useful frameworks, and suggesting doable solutions. Achieving sustainable pharmaceutical practices that support worldwide environmental and public health objectives requires sustained research, policy development, and international cooperation. The area needs to keep developing and implementing sustainable methods like green chemistry to decrease environmental harm and improve sustainability. Furthermore, collaborations among academia, industry, and international organizations are essential to progress and interchange effective strategies.

## 1 Introduction

Pharmaceutical science has a long and successful history of improving human health. Pharmaceuticals, chemical substances with specific biological effects, are essential in modern medicine, playing a crucial role in preventing, treating, and managing a wide range of diseases, thereby saving lives and improving the quality of life.^1^ However, increasing the use of drugs will increase the growing global concern of the effect of pharmaceutical production, usage, and disposal on the environment.^2^ Pharmaceuticals have a negative effect on the environment, contributing to the pollution of water sources and soil, which in turn harms wildlife. During the manufacturing process, some industries clear the chemicals improperly, releasing them into the environment, leading to contaminated drinking water and affecting microbial communities in the soil. Additionally, the continuous release of these compounds can affect wildlife and ecosystems. For example, diclofenac has been found to induce renal failure in vultures that ingested cattle carcasses treated with this drug.^3^, ^4^, ^5^ This environmental contamination poses a serious threat to public health. Inappropriate disposal of unused medications, such as flushing them down the drain, can lead to pharmaceuticals entering our drinking water. Therefore, long-term exposure to these compounds can negatively affect our health.^6^ Furthermore, this contamination can contribute to bacterial antibiotic resistance as these drugs in the environment expose bacteria and promote resistance development.^4^

Focusing on this issue is important for a healthy future for both public health and the environment. Overcoming these challenges requires a multidisciplinary approach, including developing sustainable manufacturing practices, promoting eco-friendly drugs, improving education on proper disposal and antibiotic use, and implementing strict regulations on the pharmaceutical lifecycle.^7^ Regardless of growing awareness, research gaps remain, especially about the long-term ecological effects and the level of global contamination. Additionally, there is no strict regulation enforcing sustainable manufacturing practices across industries.

Several countries have developed policies and regulations to cut the negative effects of pharmaceuticals on the environment, including the United States, Canada, the European Union (EU), and India. In the US, agencies like the Environmental Protection Agency (EPA) and the Drug Enforcement Administration (DEA) handle discarding pharmaceutical waste; however, there are no standardized procedures across the states. Canadian regulations are less stringent, with guidelines decided by individual municipalities. The EU has launched initiatives like the European Green Deal and the Zero Pollution Action Plan to address pharmaceutical pollution collectively. In India, the Central Pollution Control Board (CPCB) enforces the Zero Liquid Discharge Policy, requiring ongoing wastewater monitoring and encouraging pharmaceutical industries to achieve zero liquid discharge. While these regulations have been effective in reducing contamination and raising public awareness, they face limitations such as inconsistent implementation, inadequate monitoring, knowledge gaps about pharmaceutical toxicity, and economic constraints in developing countries.^8^, ^9^, ^10^ There is a clear need for new regulations and policies promoting sustainable medication use. These regulations should include risk assessments for new pharmaceuticals concerning environmental risks and set up global ethical standards for pharmaceutical waste management.

Assessing sustainability within the context of pharmaceuticals has been an important research agenda for scholars intending to appraise policy efficiency. For instance, Veleva et al. proposed indicators that quantify environmental sustainability which guide the evaluation of the degree of conformity with regulatory objectives of pharmaceutical firms. Such indicators may include waste recycling and management, energy conservation, and emissions and discharges control, all of which form the basic framework of compliance measures.^11^

An effective account of the impact of antibiotic-contaminated effluents from pharmaceutical industries on aquatic systems was made by Bielen et al..^12^ Their study focused on the urgency of having credible policies to control waste management because residues of antibiotics were found to be partly linked to antimicrobial resistance.^12^ In the same way, Wang et al.^13^ discussed antibiotic residues in water pollution, especially from wastewater from treatment plants and pharmaceutical factories, to seek better technologies in waste treatment and stringent measures from authorized enforcement for the pollutants.^11^ Both studies^12^^,^^13^ highlight that regulations such as the European Union's Water Framework Directive (WFD) and the U.S. Clean Water Act (CWA) govern the discharge of damaging materials, including pharmaceutical deposits, into the environment. These programs set strict contamination limits for effluents. However, enforcement is frequently inconsistent due to inadequate monitoring, regulatory gaps, and resource constraints, particularly in regions with high industrial activity. Weak enforcement enables the continued release of these substances, underscoring the need for stronger governance and more stringent regulatory oversight.^12^^,^^13^ Milanesi et al. further elaborated on these gaps, stating that despite the adoption of sustainability benchmarks by organizations, the absence of standard regulatory environments worldwide threatens their efficiency. This clearly shows the need for policy convergence in international markets to enhance sustainability practices.^14^

Achieving sustainable medicine use requires cooperation among different stakeholders, including governments, pharmaceutical companies, healthcare providers, and the public. Governments could set global guidelines for eco-friendly practices that encourage the reuse of medications and the proper disposal of unused and expired drugs.^15^ Pharmaceutical industries can develop sustainable practices, such as recyclable drug delivery systems and take-back programs.^16^ Healthcare providers can educate patients on proper disposal, prescribe medications tailored to individual needs to reduce waste, and use technology like electronic health records to track medication usage.^17^ The public can follow government instructions on disposing of expired medications, contributing to a healthier environment.^18^^,^^19^

To mitigate gaps in regulatory compliance, auditing tools such as life cycle assessments (LCAs) have been implemented. Mata et al. demonstrated how LCAs assess the environmental effects of pharmaceutical processes systematically.^20^ However, the adoption of LCAs remains limited due to information and funding challenges, particularly among small-scale pharmaceutical companies. Extending the use of such tools through regulations and new training could radically improve compliance.^21^ Additionally, incentives like tax exemptions or grants could support companies in adopting sustainable practices.^22^

Other studies have established that innovation plays a strategic role in improving sustainability practices within the pharmaceutical industry. Blum-Kusterer and Hussain^23^ emphasized the need for appropriate governmental regulations to promote research and development for sustainability programs.^23^ Regulatory programs, such as reverse logistics, highlighted by Narayana et al.,^24^ promote proper recycling and disposal of unused medications, which minimize the negative impact on the environment and foster a circular economy.^24^ Collaborative governance models that involve regulators, industry stakeholders, and academic researchers, as suggested by Donkor et al., can help address regional disparities in regulatory compliance and promote sustainable development objectives globally.^25^

The current study aimed to investigate how regulatory frameworks and policies are used to support the use of sustainable medicines within the pharmaceutical sector. Its three specific aims were to evaluate the extent to which existing regulations and policies effectively promote environmental sustainability; to identify potential deficiencies in current approaches; and to suggest new policy measures to improve the sustainability of pharmaceutical practices.

## 2 Methods and materials

### 2.1 Database

Of the various reputable online databases available, the current study selected Scopus to search for publications addressing the role of regulatory frameworks and policies in promoting sustainable medicines. Scopus has been widely acknowledged to be among the most comprehensive online databases available for bibliometric researchers.^26^^,^^27^

Scopus was created by Elsevier, a renowned international publisher of scientific journals, and was selected in this study over databases such as the Web of Science (WoS), Medline, and Google Scholar for several reasons.^28^, ^29^, ^30^, ^31^ 1) Scope and quantity of materials stored: Scopus makes available an enormous amount of research literature from scholars of multiple disciplines and types (e.g., peer-reviewed journals, conference proceedings, patents, book series, trade publications). Hence, it is recommended for research seeking to analyze research trends across more than one discipline. (2) Quality of selection process: A painstaking screening and selection process is applied to all research literature before inclusion in Scopus; consequently, the data are deemed to be high-quality. Moreover, a team of content specialists monitors the quality of data in the included literature and, where necessary, updates it to maintain accuracy. (3) Complexity of searches: Users are not restricted by search mode, as Scopus enables searches by author, publication, affiliation, keyword, and citation. (4) Provision of popular citation metrics: To enable bibliometric studies to assess the impact and influence of the authors and works included in the database, Scopus makes available citation counts, h-index, and co-citation analysis. (5) Easy integration: Data visualization and research analytics tools, as well as other software programs, can all be integrated, facilitating the analysis, value, and presentation of bibliometric data. (6) Breadth of coverage: As Scopus includes most of the journals indexed in WoS, MEDLINE, and Embase, it can be regarded as among the broadest of bibliometric analysis resources.

### 2.2 Search strategy

Using Scopus's “advanced search” feature, the researchers drew up a list of keywords (i.e., search queries) most likely to yield research relevant to the purpose of the current study, namely, those addressing the role of regulatory frameworks and policies that promote the use of sustainable medicines. As the Scopus database is continually updated, care was taken to mitigate any related bias by extracting and exporting all documents on a single day (July 3, 2024). The search strategy is described in more detail in the following section.

TITLE ((“sustainable medicines” OR “sustainable pharmaceuticals” OR “green pharmacy” OR “pharmaceutical industry” OR “pharma sector”) AND (sustainability OR “environmental impact”)).

### 2.3 Data extraction

This bibliometric review study aimed to use all articles identified as containing the selected keywords in their titles. The inclusion criteria were that the articles must be published in English-language journals between 2000 and 2024. Following recommendations in the literature,^32^, ^33^, ^34^, ^35^, ^36^ we searched for articles whose titles alone – rather than titles and abstracts – contained the selected keywords because this strategy reduces the number of documents returning false-positive results. To be clear, searching within both titles and abstracts would have likely yielded many false positives because the principle focus of the studies thus identified would be on matters other than the regulatory frameworks and policies supporting sustainable medicines that are the focus of the current review. Further points to note in the research approach adopted are that the asterisk (*) was employed as a wildcard, and quotation marks (“”) were used to pinpoint specific terms or phrases. We excluded errata and retracted documents. These search criteria yielded an initial total of 44 articles, which we subjected to manual screening procedures, namely, reading the complete texts and assessing content to eliminate those irrelevant to our research process, thus enhancing the overall quality of the data to be included in our review.

### 2.4 Validation of the search strategy

The next step after the measures outlined above was to ensure false positives were eliminated from the list of articles identified as potentially relevant. To this end, the researchers analyzed the 20 publications that were most frequently cited in the returned articles to assess whether they were indeed relevant to the topic at hand. Specifically, two bibliometric experts evaluated the titles and abstracts of these 20 publications to confirm they did not include any false positives, after which this element in the search process was considered as finished.

Furthermore, a correlation test was run. The aims of this test were first, to determine whether the data retrieved by the search process correlated with the findings of the ten scholars deemed most active in the research area; and second, to identify false negatives, namely, any relevant publications, data, or research gaps that the initial search had missed, which would be indicated by a lower correlation coefficient. The results of a correlation test enable researchers to modify their search by, for example, adjusting keywords, criteria, search operators, and search fields to enhance the quality and validity of the results. The test returned a correlation of *r* = 0.871, which is considered strong and statistically significant (*p* < 0.001). Hence, the search query was deemed accurate and valid. This validation method has been used in the literature.^37^^,^^38^

As can be seen from the descriptions above, an exhaustive and rigorous approach was taken in this study to ensure accuracy. Moreover, further credibility and validation are given to the findings of this review by the participation of bibliometric experts and the use of a correlation test. It can thus be said that the authors of this study made all possible attempts to ensure this review and its findings were of high quality and reliability (Supplementary data).

### 2.5 Data export and data management (data analysis and visualization)

Once the search strategy described above had been executed, all the data retrieved were exported to Microsoft Excel in “csv” format. The main focus of the analyses performed for this study were the percentages and frequencies of publication; however, the retrieved data also concerned the document types, funding agencies, citations, and journal names of the identified articles and, for each individual article, the titles, abstracts, and authors' countries of origin and institutional affiliations.

We then proceeded to create network maps, utilizing VOSviewer software version 1.6.20 (Leiden University, Leiden, The Netherlands), to better visualize the relations between the terms appearing in article titles or abstracts and international collaborations. We also sought to predict future research “hotspots” by using the same software program to generate scientifically grounded knowledge networks revealing the progress of distinct fields of research. VOSviewer allows the user to deploy co-occurrence analysis to group terms into multiple clusters, each with a unique color. Indeed, it can be preferable to generate a cluster analysis of research hotspots than create a co-occurrence network comprising the terms used in titles or abstracts to visualize and reveal developing trends.

## 3 Results

### 3.1 Overview of the retrieved publications

A total of 43 publications on Sustainable Medicines Use were found between 2000 and 2024, namely 29 (67.4 %) Articles, 6 (14 %) Book chapters, 3 (7 %) Reviews, 1 (2.3 %) Notes (Table 1).

**Table 1:** Types of publications on sustainable medicines use.

Document type	Count	Percentage
Article	29	67.4
Book chapter	6	14
Conference paper	4	9.3
Review	3	7
Note	1	2.3
Total	43	100 %

### 3.2 Investigating trends in growth and productivity

**Fig. 1:**
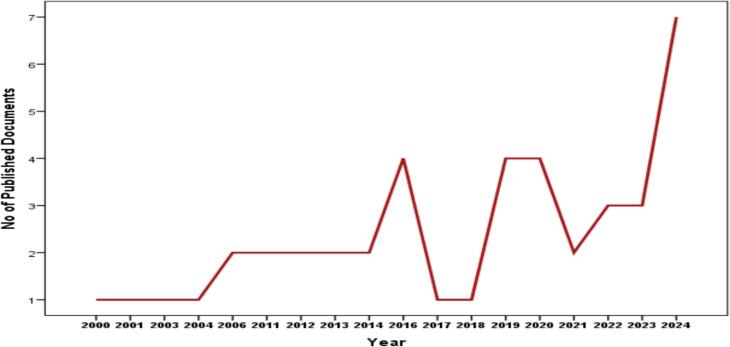
Publication trends on sustainable medicines use from 2000 to 2024.

Fig. 1 shows the trend in the number of published documents on sustainable medicines use from 2000 to 2024, revealing an overall growth with notable fluctuations. Initial years (2000−2010) saw low publication numbers, with either 1 or 2 documents per year. A moderate increase occurred between 2011 and 2015, peaking at 4 publications in 2014, followed by a drop to 1 in 2015. Significant fluctuations are observed from 2016 to 2021, with peaks in 2016 and 2019 (4 publications each) and dips in 2017 and 2021 (1 or 2 publications). A substantial surge in research activity is evident from 2022 onwards, culminating in 7 publications in 2024, indicating growing interest and focus on sustainable medicines use. Key years of intensified research include 2014, 2016, 2019, and 2024, reflecting periods of heightened academic activity in this field.

### 3.3 Evaluation of leading countries in publication output

**Fig. 2:**
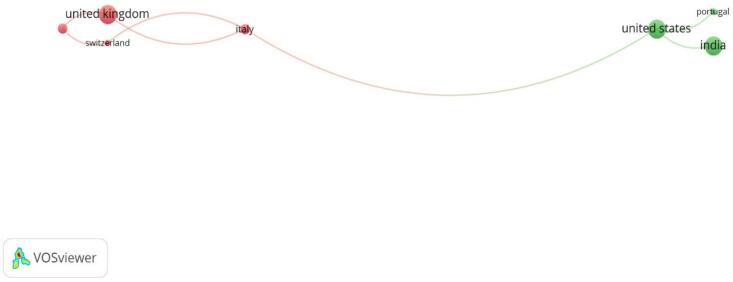
A visualization map was generated using VOS viewer software version 1.6.20, illustrating the international research collaboration network with a minimum contribution of 5 documents per country as the threshold (*n* = 24). Countries that are closely connected and have dense relationships exhibit robust scientific collaboration. Conversely, countries on the periphery with weak connections to central countries demonstrate limited international research collaboration.

The visual representation from VOSviewer illustrates the leading countries in publication output on sustainable medicines use. The map highlights key countries such as the United Kingdom, United States, India, Italy, Portugal, and Switzerland, indicating their collaborative relationships and publication volumes. The United Kingdom and United States emerge as prominent contributors, connected with other nations, suggesting strong research networks and high productivity in this field. Italy, Portugal, and India also show significant contributions, reflecting their involvement in sustainable medicines research. This map underscores the global distribution of research efforts and the interconnectedness among these countries in advancing sustainable medicines use (Fig. 2 & Table 2).

**Table 2:** Countries' contributions to scientific research on sustainable medicines use.

Country	Documents	%	Citations	Total link strength
India	5	11.63 %	60	1
United Kingdom	5	11.63 %	114	2
United states	5	11.63 %	139	3
Germany	4	9.3 %	3	0
Brazil	2	4.65 %	1	0
France	2	4.65 %	0	0
Greece	2	4.65 %	7	0
Italy	2	4.65 %	87	3
Romania	2	4.65 %	4	2
Sweden	2	4.65 %	44	0

### 3.4 Analysis of top prolific institutions

**Fig. 3:**
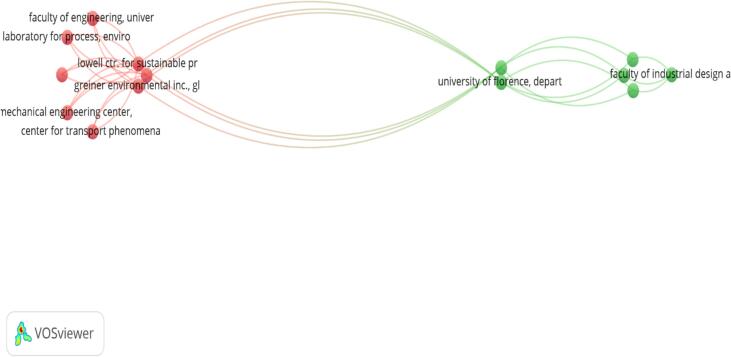
A visualization map was generated using VOS viewer software version 1.6.20, illustrating the international research collaboration network with a minimum contribution of 5 documents per institution as the threshold (*n* = 24). Institutions that are closely connected and have dense relationships exhibit robust scientific collaboration. Conversely, institutions on the periphery with weak connections to central institutions demonstrate limited international research collaboration.

A total of 92 institutions have been involved in research on Sustainable Medicines Use. Fig. 3 illustrates the network of top prolific institutions in the field of sustainable medicines use. Key institutions such as the Faculty of Engineering and the Laboratory for Process and Environmental Engineering, Lowell Center for Sustainable Production, Greiner Environmental Inc., and the University of Florence are prominently featured, indicating their significant contributions to research in this area. The University of Florence and the Faculty of Industrial Design are shown to have strong collaborative connections, highlighting their active roles in advancing sustainable medicines. The map underscores the importance of interdisciplinary and inter-institutional collaborations in driving research productivity and innovation in sustainable medicines use.

### 3.5 Investigating high-output journals

**Fig. 4:**
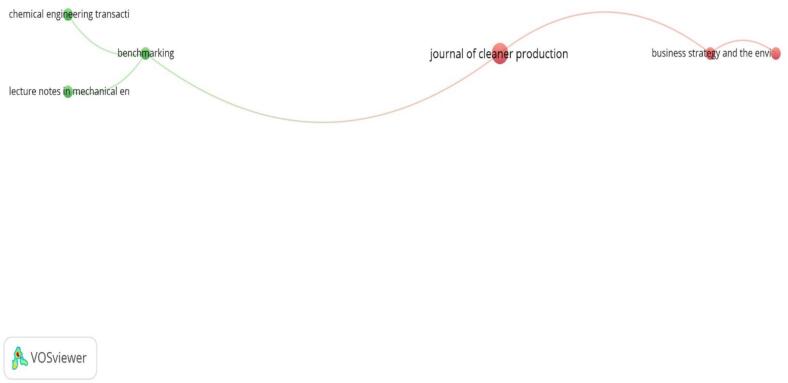
A visualization map was generated using VOS viewer software version 1.6.20, illustrating the international research collaboration network with a minimum contribution of 5 documents per institution as the threshold (*n* = 42). Journals that are closely connected and have dense relationships exhibit robust scientific collaboration. Conversely, Journals on the periphery with weak connections to central Journals demonstrate limited international research collaboration.

Fig. 4 illustrates the high-output journals in the field of sustainable medicines use. Key journals such as the “Journal of Cleaner Production,” “Business Strategy and the Environment,” “Chemical Engineering Transactions,” “Benchmarking,” and “Lecture Notes in Mechanical Engineering” are prominently featured. The “Journal of Cleaner Production” and “Business Strategy and the Environment” stand out, indicating their significant contributions and central roles in disseminating research on sustainable medicines. These journals are connected, highlighting the interdisciplinary nature of the field and the broad range of topics covered, from engineering and benchmarking to business strategy and environmental impacts. This map underscores the importance of these journals in advancing research and knowledge in sustainable medicines use.

### 3.6 Analysis of citations

According to the citation analysis, the retrieved articles have been cited an average of 22.26. The range of citations was between 0 and 247. About 13 of the retrieved articles had no citations, while 10 received 20 or more citations. The top ten most-cited articles received a total of 863 citations.^11^, ^12^, ^13^, ^14^^,^^20^^,^^23^^,^^24^^,^^25^^,49,^^29^ The total number of citations for these articles on sustainable medicines use ranged between 247 and 20 (Table 3).

**Table 3:** The top 10 articles with the highest citations in studies concerning sustainable medicines use.

Author	Title	Year	Source title	Cited by
Bielen et al.^12^	Negative environmental impacts of antibiotic-contaminated effluents from pharmaceutical industries	2017	*Water Research*	247
Wang et al.^13^	Antibiotic residues in wastewaters from sewage treatment plants and pharmaceutical industries: Occurrence, removal and environmental impacts	2021	*Science of the Total Environment*	127
Veleva et al.^11^	Indicators for measuring environmental sustainability: A case study of the pharmaceutical industry	2003	*Benchmarking*	123
Milanesi et al.^14^	Pharmaceutical industry riding the wave of sustainability: Review and opportunities for future research	2020	*Journal of Cleaner Production*	84
Blum-Kusterer & Hussain^23^	Innovation and corporate sustainability: An investigation into the process of change in the pharmaceuticals industry	2001	*Business Strategy and the Environment*	83
Al-Awamleh et al.^39^	The effect of green supply chain on sustainability: Evidence from the pharmaceutical industry	2022	*Uncertain Supply Chain Management*	58
Federsel^40^	In search of sustainability: process R&D in light of current pharmaceutical industry challenges	2006	*Drug Discovery Today*	43
Narayana et al.^24^	Market dynamics and reverse logistics for sustainability in the Indian Pharmaceuticals industry	2019	*Journal of Cleaner Production*	40
Mata et al.^20^	Lca tool for sustainability evaluations in the pharmaceutical industry	2012	*Chemical Engineering Transactions*	38
Donkor et al.^25^	The supply chain integration – Supply chain sustainability relationship in the UK and Ghana pharmaceutical industry: A stakeholder and contingency perspective	2021	*Transportation Research Part E: Logistics and Transportation Review*	20

### 3.7 Top ten articles

The top ten influential papers on sustainable medicine use emphasize key challenges, current policies, and necessary measures to improve sustainability in the pharmaceutical industry. Major concerns include significant environmental pollution from pharmaceutical off products in wastewater, as highlighted by Bielen et al.^12^ and Wang et al.,^13^ inefficiencies in resource use during production,^11^^,^^40^ and difficulties in supply chain integration.^24^^,^^25^

Current regulations emphasize environmental risk assessments (ERAs), with the EU's revised pharmaceutical legislation requiring ERAs for new medicines to mitigate environmental risks.^12^^,^^13^ The World Health Organization (WHO) has also called for regulatory reforms to reduce the environmental impact of medical products while ensuring safety and efficacy standards.^14^ Initiatives, like the European Green Deal and EFPIA's sustainability proposals, advocate for CO₂ reduction targets, renewable energy adoption, and increased use of recycled packaging materials.^14^^,^^39^

Despite these efforts, enforcement remains sporadic, especially in monitoring emissions from pharmaceutical production.^12^^,^^13^ Addressing these gaps requires stronger enforcement mechanisms, global alignment of sustainability regulations, and greater investment in green technologies such as eco-friendly drug formulations and renewable energy sources. Additionally, fostering collaboration between regulators and industry stakeholders is crucial for driving innovation and ensuring the pharmaceutical sector meets global sustainability goals while maintaining access to safe and effective medicines.^11^^,^^14^^,^^23^^,^^24^^,^^25^^,^^39^

### 3.8 Co-occurrence analysis

**Fig. 5:**
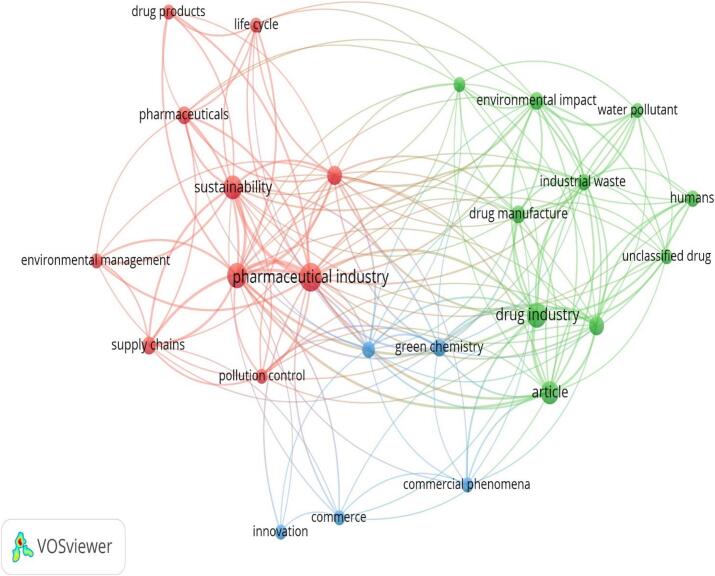
A cluster map was generated based on the analysis of terms found in titles or abstracts. The circle's size represents how often the terms appear, while various colors indicate different clusters. This map was created using VOS viewer software version 1.6.20.

The visualization of frequently occurring terms in the titles and abstracts of collected documents, appearing at least 3 times, revealed 25 terms categorized into three distinct-colored clusters (red, green and blue) representing the top three research priority topics (see Fig. 5). Red Cluster (Pharmaceutical Industry and Sustainability): This cluster focuses on terms such as “pharmaceutical industry,” “sustainability,” “pharmaceuticals,” “life cycle,” “drug products,” “environmental management,” “supply chains,” and “pollution control.” It highlights the emphasis on the pharmaceutical industry's role in sustainability, addressing lifecycle management, environmental impact, and sustainable supply chains.

Green Cluster (Environmental Impact and Industrial Processes): The green cluster includes terms like “environmental impact,” “industrial waste,” “drug manufacture,” “drug industry,” “water pollutant,” “humans,” and “unclassified drug.” This cluster emphasizes the environmental consequences of industrial processes, focusing on waste management, pollution control, and the impact on human health and ecosystems.

Blue Cluster (Innovation and Commercial Phenomena): The blue cluster comprises terms such as “green chemistry,” “innovation,” “commerce,” “commercial phenomena,” and “article.” This cluster highlights the importance of innovation in green chemistry and sustainable practices, alongside the commercial aspects and economic implications of implementing sustainable solutions in the pharmaceutical industry. Overall, this visualization provides a comprehensive overview of the key themes and research priorities in the field of sustainable medicines use, emphasizing the interconnectedness of industrial practices, environmental impact, and innovative solutions for sustainability.

### 3.9 Analysis of future research directions

**Fig. 6:**
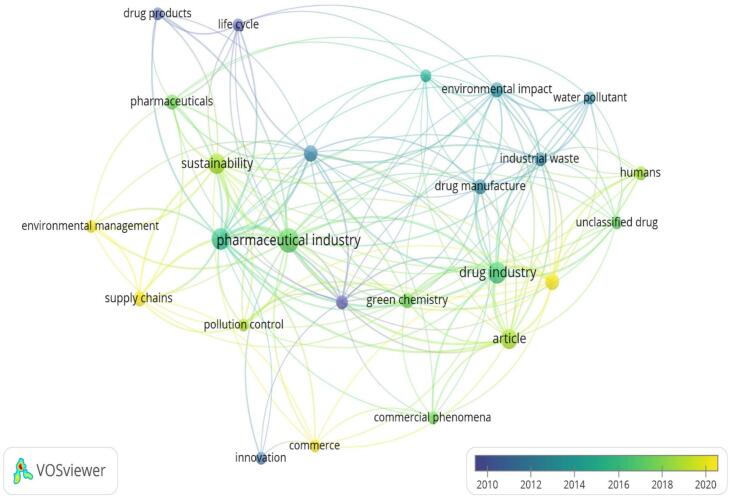
A network visualization map was generated to analyze the frequency of terms appearing in titles. Blue represents terms that appeared earlier, while yellow indicates later appearances. This map was created using VOS viewer software version 1.6.20.

Fig. 6 depicts an overlay visualization where VOS viewer was utilized to color terms based on their publication year. The overlay visualization created using VOSviewer highlights temporal trends in sustainable medicines use research, with terms color-coded from blue (around 2010) to yellow (around 2020) to indicate their prominence over time. Early research (blue shades) focused on terms like “drug products,” “life cycle,” and “environmental management,” reflecting initial efforts to understand the lifecycle impacts of pharmaceuticals and early environmental management practices within the industry. Mid-period terms (green shades) such as “pharmaceutical industry,” “sustainability,” “pollution control,” and “drug manufacture” gained attention from 2013 to 2016, indicating a focus on integrating sustainability practices and addressing pollution control within the pharmaceutical sector. More recent terms (yellow shades), including “unclassified drug,” “drug industry,” “innovation,” and “commercial phenomena,” suggest a shift towards exploring new drug categories, innovative approaches, and the commercial aspects of sustainability. Future research directions are likely to delve deeper into innovative methods and sustainable chemical practices (green chemistry), reflecting an emphasis on developing greener processes and products. Economic implications and market dynamics associated with sustainable practices will also be a focal point, as indicated by the prominence of commerce-related terms. Additionally, ongoing concerns about ecological and human health impacts will drive research on mitigating industrial waste and pollution. The interconnectedness of terms across clusters underscores the interdisciplinary nature of this research area, suggesting that future studies will integrate insights from environmental science, chemistry, economics, and public health to advance sustainability in the pharmaceutical industry.

## 4 Discussion

This study highlights the growing interest in literature towards the sustainable use of pharmaceuticals from 2000 to 2024. We analyzed 43 publications, including 67.4 % articles, 14 % book chapters, and a few reviews and conference papers. The findings revealed a prominent level of interest, especially in recent years. The several types of documents illustrate various aspects of sustainability in pharmaceuticals, from technical studies to policy discussions.

There is an overall upward trend in the publication rate on sustainable medicines use from 2000 to 2024, with some notable fluctuations. While in the first years (2000–2010) there were low publication numbers, with only 1 or 2 documents per year, there has been a gradual increase, culminating in 7 publications in 2024. Similar findings were reported by Milanesi et al.,^14^ who analyzed 59 articles published between 2000 and 2019 and found that research on pharmaceutical sustainability began to increase in 2016.^14^ Another study highlighted the importance placed on environmental sustainability in the pharmaceutical sector and the focus on implementing practices that support this goal.^41^

The United Kingdom and the United States have strong research networks and high publication output. Other countries like India and Portugal also show significant contributions, indicating robust international collaboration. This confirmation of results highlights the increasing significance and attention to sustainability in the pharmaceutical sector, showing a collaborative approach to challenge environmental effects and enhance sustainable methods. Our results revealed the interdisciplinary nature of the research, with ninety-two institutions taking part, such as the Faculty of Engineering, Lowell Centre for Sustainable Production, and the University of Florence. Journal of Cleaner Production,^14^ Business Strategy and the Environment,^23^ Chemical Engineering Transactions,^20^ Benchmarking,^39^ and Lecture Notes in Mechanical Engineering were identified as high-output journals central to the research findings.

The analysis of terms in the titles and abstracts shows three primary research areas: Sustainability in Pharmaceutical Industry, Environmental Impact on Industrial Processes, and Commercial Phenomena Innovation. These groupings show how industrial practices, environmental effects, and creative sustainability solutions are all connected. The overlap visualization shows a change over time in research focus, with older studies focusing on lifecycle effects and environmental management, mid-period research including sustainability practices,^39^ and recent studies examining new drug categories, innovative approaches, and the commercial side of sustainability.

The EU has been the leading party in regulating the concept of sustainability with regards to pharmaceuticals. The EU's Green Deal and the Zero Pollution for a Healthy Europe communication focus on cutting emissions, limiting pharmaceutical waste, and promoting green chemistry and innovation. These frameworks require improved containment of manufacturing emissions and wastewater effluent, thus prior elimination/minimization of pharmaceuticals.^11^ Such extensive policies compel pharmaceutical companies to adopt sustainable practices and contribute to innovation and sustainable manufacturing technologies, as pointed out by Milanesi et al..^14^

Policies implemented in the EU are worthy of imitation in other regions. For instance, in Italy, understandings and use of green supply chain practices have gained considerable advancement. Al-Awamleh et al.^39^ pointed out that nature-based solutions, such as offering subsidies for better means of transport and for recycling in pharmacy, helped bring down the carbon footprint.^39^ Likewise, the United Kingdom has employed reverse logistics, which entails the collection of medicines past their shelf life or untouched medicines for proper disposal or recycling. Donkor et al.^25^ noted that such regulatory requirements decrease the environmental impact and increase corporate responsibility in the UK pharmacy industry.^42^

Current bibliometric review revealed one major shortcoming: the lack of internationally approved guidelines on the management of pharmaceutical waste. Wang et al.^30^ and Bielen et al.^12^ noted that the absence of consistency causes high variability in waste management trends, including its handling and disposal, presenting high variability in environmental effects across different regions.^43^ While the EU sets high standards for wastewater treatment, many developing countries do not have the facilities and resources to follow suit.

Cognition and synergy with members from other disciplines are useful when designing the next generation of pharmaceutical practices that can be more sustainable. Blum-Kusterer and Hussain^23^ concluded that innovation and sustainable development initiatives should be framed by regulations.^44^ However, to date, such partnerships lack coordination and a common purpose, limiting their impact.

Rewards on green chemistry and sustainable supply chain innovation can promote development. As reflected in the survey by Blum-Kusterer and Hussain^23^ and Federsel,^40^ sustainability reporting (SR) reinforced that initiatives like tax credits or grants for environmentally sound research and development (R&D) compel firms to undertake sustainable initiatives. They ensure that all economic processes are tied to sustainability goals, making achieving objectives easier.^45^ Training and government support for small and medium-sized enterprises (SMEs) are vital in progressing their sustainability efforts.^46^

The results of this study have multiple implications for the pharmaceutical sector. A noticeable shift is happening towards incorporating sustainability into different areas of pharmaceutical manufacturing and delivery. The area needs to keep developing and implementing sustainable methods like green chemistry to decrease environmental harm and improve sustainability. Furthermore, collaborations among academia, industry, and international organizations are essential to progress and interchange effective strategies.

Overall, there is an increased number in research that concerns the sustainable use of medication, it was done in different countries and institute. Focusing on multidisciplinary and shared methods highlights the significance of sustainability in the pharmaceutical sector due to its complexity. Future studies should further investigate innovative approaches and the economic and ecological consequences of sustainable methods to progress the field and support worldwide sustainability goals.

The current paper presents the first bibliometric review of the emerging topic of the role of regulation and policy in supporting sustainable medicine and offers a broad and multifaceted view of related research trends and hotspots. In particular, this study is unique in three ways: (1) it uses Scopus, the most comprehensive database available; (2) it makes comprehensive use of terms associated with the role of regulatory frameworks and policies in supporting the use of sustainable medicines; and (3) it applied a global scope to the search query. It must nonetheless be acknowledged that there are some limitations to the findings of this research. First, our analysis is based on papers retrieved only from the Scopus database which, despite containing most published research papers in the area of interest, may not have contained some research found in other databases (e.g., PubMed and WoS). Second, we may not always have correctly identified the geographical origin of the papers included from the affiliation information supplied by their authors. Third, only papers written in English were considered. Fourth, factors external to the quality of the research itself may have influenced citation analyses, for example, journal impact factors and self-citations.

## 5 Conclusion

This bibliometric review offers a detailed overview of how research into the use of sustainable medicines has developed as well as its current state. We have identified the most important contributors, publications, and emergent research trends, and we have sought to underline that the research field is both interdisciplinary and collaborative in nature. This study offers a thorough analysis of the legislative and governmental strategies promoting the use of sustainable medicine. It offers important insights for promoting sustainability in the pharmaceutical industry by pointing out gaps, defining useful frameworks, and suggesting doable solutions. Achieving sustainable pharmaceutical practices that support worldwide environmental and public health objectives requires sustained research, policy development, and international cooperation.

## Funding

No funding was used to assist in the preparation of this study.

## Ethical approval

Current study did not involve any interactions with humans, approval from the Ethics Committee was not required.

## Ethics approval and consent to participate

Not applicable.

## CRediT authorship contribution statement

**Ammar Abdulrahman Jairoun:** Writing – review & editing, Writing – original draft, Visualization, Validation, Software, Methodology, Investigation, Formal analysis, Data curation, Conceptualization. **Sabaa Saleh Al-Hemyari:** Writing – review & editing, Writing – original draft, Supervision, Software, Funding acquisition, Formal analysis, Conceptualization. **Moyad Shahwan:** Writing – review & editing, Supervision, Software, Resources, Methodology, Funding acquisition. **Sahab Alkhoujah:** Writing – original draft. **Faris El-Dahiyat:** Writing – review & editing, Software, Investigation, Data curation, Conceptualization. **Ammar Ali Saleh Jaber:** Writing – original draft, Software, Resources. **Sa'ed H. Zyoud:** Writing – review & editing, Writing – original draft, Visualization, Validation, Supervision, Investigation.

## Declaration of competing interest

All authors declare that they have no conflict of interest.

## Data Availability

The original contributions presented in the study are included in the further inquiries can be directed to the corresponding authors.
